# Mapping of the venomous stingrays of the *Potamotrygon* genus in the Tietê River, São Paulo Sstate, Brazil

**DOI:** 10.1590/0037-8682-0216-2022

**Published:** 2022-11-04

**Authors:** Isleide Saraiva Rocha Moreira, Vidal Haddad

**Affiliations:** 1 Universidade Estadual Paulista, Faculdade de Medicina Veterinária e Zootecnia, Botucatu, SP, Brasil.; 2 Universidade Estadual Paulista, Faculdade de Medicina de Botucatu, Botucatu, SP, Brasil.

**Keywords:** Freshwater Stingrays, Ulcers, Bites and stings, Venomous animals

## Abstract

**Background:**

Freshwater stingrays are fish that have adapted to the rivers and lakes in South America. The expansion of the Potamotrygonidae family in the Paraná River began after the damming of the Sete Quedas Falls, reaching the mouth of the Paranapanema and Tietê rivers approximately 20 years ago via the locks of the hydroelectric power plants. They are not aggressive animals; however, they have one to four stingers on their tails covered by a venom-producing epithelium and can cause severe envenomation in fishermen and bathers if stepped on or manipulated.

**Methods:**

We conducted a descriptive, retrospective, and prospective study by monitoring the fishing of the Potamotrygon genus in the lower Tietê River, mapping the location of the rays as a fishery product of professional fishermen and/or recording images of the fish caught.

**Results:**

Sixteen stingrays of the Potamotrygon genus were mapped by monitoring fishermen's fish products in the extensive area between the municipalities of Pereira Barreto and Buritama, São Paulo state.

**Conclusions:**

The lower Tietê River is fully colonized by freshwater stingrays and this expansion likely continues upstream, reaching various sub-basins of the river. The advancement of these venomous fish in areas where they did not exist previously requires education programs and interaction with the community to avoid serious injuries in bathers and fishermen and the unreasonable extermination of the animals.

## INTRODUCTION

**FIGURE 1:**
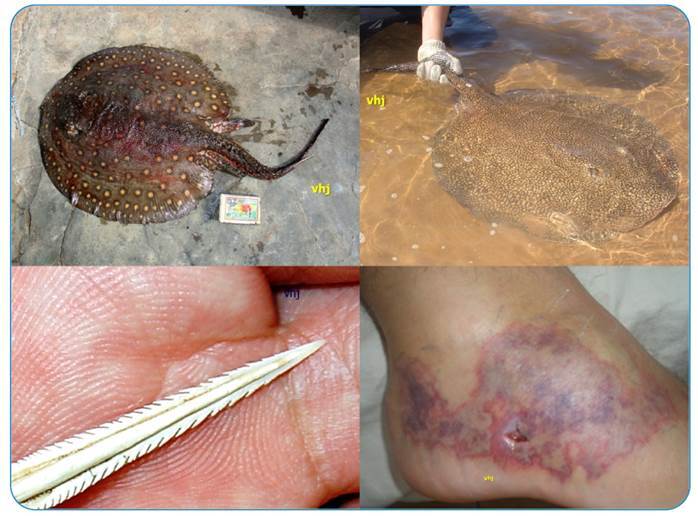
Freshwater stingrays of the Potamotrygonidae Family: **(A)** *Potamotrygon falkneri* (Castex & Maciel 1963); **(B)** *Potamotrygon motoro* (*P. amandea*) (Müller & Henle 1841). Note the serrated stingers and the envenomation caused by a freshwater stingray in a bather in the Paraná River region.

Freshwater stingrays of the Potamotrygonidae family are Elasmobranchii adapted to freshwater environments and are distributed in the main hydrographic basins and rivers of South America^1^. They are considered venomous animals because they have stingers covered by venom-producing epithelium on their tails (Figure 1) and defend themselves by whipping their tails when they feel threatened or stepped on, causing serious injuries and envenomation via the stingers^2^ ^-^ ^6^. These serrated stingers are rigid, made of dentin, and covered by a mucus rich in at least 18 toxins^2^ ^,^ ^4^ ^,^ ^6^. The venom of stingrays, when injected, causes violent pain initially, and in later stages provokes intense inflammation with edema, erythema, and deep and extensive cutaneous necrosis (Figure 1)^2^. As these fish are habitually semi-buried in sandy and muddy bottoms, envenomation occurs in bathers who use the artificial beaches created by the damming of river waters in various regions of the country^4^.

The characteristics of the injuries are similar to other stingray species in South America. They most often affect the feet, legs, and hands, with an increase in cases occurring in the summer. Because the venom is thermolabile and has a vasoconstrictor effect, the first step in immediate care is to place the injured site in hot water at 50 °C, which causes vasodilation of blood vessels and relieves the pain. The next measure is to refer the victim to a health service for cleaning the wound, applying dressings, antibiotic therapy, and tetanus vaccination^2^ ^-^ ^4^ ^,^ ^4^ ^,^ ^6^ ^,^ ^12^. Many fishermen use popular treatments on the wound site without success, such as urine, herbs, oils, and gasoline^6^ ^,^ ^7^ ^,^ ^6^ ^-^ ^12^. There is no antivenom serum available for envenomation by freshwater stingrays^2^.

The migration of freshwater stingrays and other fish to the middle and upper Paraná River began with the damming of the Sete Quedas Falls, forming the reservoir of the Itaipu Hydroelectric Power Plant. For approximately 40 years, stingrays have used the locks for the passage of waterways in hydroelectric plants for transport, which has favored the expansion of the species^4^ ^,^ ^8^ ^,^ ^11^. In 1999, it was reported that the Paraná River above Sete Quedas (including São Paulo state) was colonized by freshwater stingrays not previously found in these regions and that this expansion would reach the Tietê River^3^ ^,^ ^12^. As the stingrays do not have natural predators in this area, they quickly spread from the lower to the upper Paraná region, reaching the mouth of the Tietê River. There is the additional risk of freshwater stingrays invading the lower Paraná, Paranaíba, and Grande rivers in Minas Gerais state.

The Tietê River begins at 1,030 m above sea level in the town of Salesópolis and travels 1,136 km inland to its mouth in the Paraná River on the border of Mato Grosso do Sul, bathing 62 municipalities in São Paulo state. The Tietê River watershed is divided into six hydrographic sub-basins: Upper Tietê, Middle Tietê, Piracicaba/Jundiaí, Tietê/Jacaré, Tietê/Batalha, and Lower Tietê (mouth of the Paraná River and the reference of this study), covering the entire São Paulo state^13^. The Tietê River is an important source of subsistence for artisanal fishermen and is of great economic importance to the state because it has a high potential to generate electricity, which allows the creation of dams to supply various regions^13^ ^,^ ^14^.

The last municipality bathed by the Tietê River in the State of São Paulo is Itapura and between 2002 and 2005, two species of stingrays of the Potamotrygonidae family (*Potamotrygon motoro* and *Potamotrygon falkneri*) were recorded in the municipality via a study with fish catches^3^ ^,^ ^12^. In places where stingrays are endemic (midwestern and northern regions of Brazil), there is a large number of envenomation and it is, therefore, important to know the current location of these fish^7^.

The objectives of this study were to evaluate the expansion of freshwater stingrays in the main river of the most populous state in the country and to measure the risk of injuries caused by stingrays in the region.

## METHODS

A bidirectional descriptive, retrospective, and prospective study was conducted to monitor the fishing of freshwater stingrays in the Tietê River of São Paulo state from 2018 to 2021, considering that the first record of a stingray in the municipality of Buritama was in 2016. This study was approved by the Ethics Committee on the Use of Animals - CEUA - Protocol 0029/2019 of the Faculty of Veterinary Medicine and Zootechnics of Botucatu - FMVZ / UNESP and the Research Ethics Committee of the Faculty of Medicine of Botucatu / UNESP, Opinion number: 3,316,728 CAAE: 12219519.6.0000.5411.

Stingrays recorded by image (photography or video) or fishing were considered between March 1st and October 31st of each year, the period released for fishing according to the Brazilian Institute of the Environment and Renewable Natural Resources Normative Instruction (2009). From November 1st to February 28th, fishing in boats with nets is prohibited for the natural reproduction of fish.

The reference municipalities for the study were Pereira Barreto, Araçatuba, Santo Antônio do Aracanguá, and Buritama, located in the lower Tietê River region, and the municipality of Novo Horizonte, located in the Tietê-Batalha region. Visits to the places of embarkation and disembarkation of fishermen were not carried out from 2020 to May 2021 due to the SARS-CoV-2 virus pandemic, following the health guidelines of the Government of the State of São Paulo and Municipalities. However, the authors monitored the capture of stingrays through telephonic contact and messaging applications and received images and videos. The fishing locations of the stingray specimens were sent by the Google Maps application (https://www.google.com/maps), but not all fishermen had a mobile phone with the GPS application, making it difficult to determine the exact location of the stingrays caught.

## RESULTS

**FIGURE 2:**
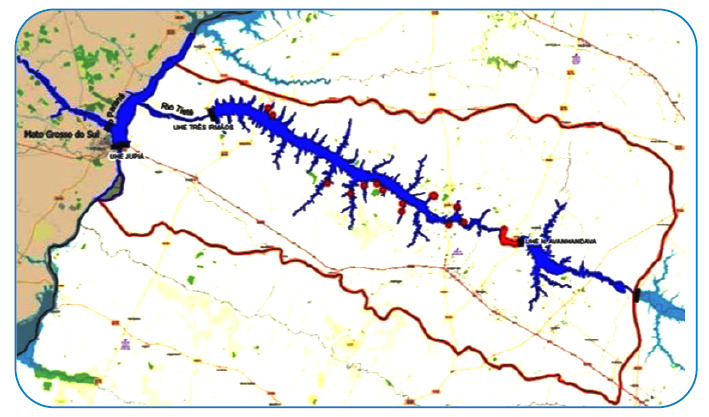
Locations with stingray captures along the initial third of the Tietê River.

**FIGURE 3:**
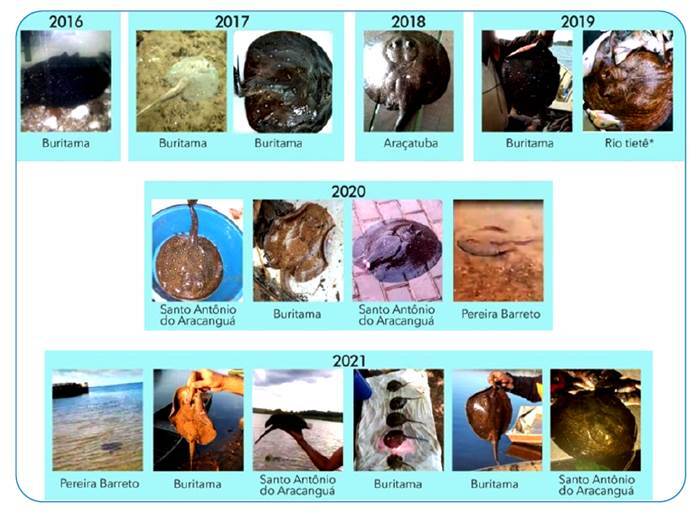
Images of stingrays captured in the lower Tietê River.

Several stingray capture points were mapped along the lower Tietê River from the municipality of Buritama to Pereira Barreto; however, in this study, only 16 points have image records of stingray capture in the period from 2016 to 2021 (Figure 2). In Buritama, the points are at a distance of 10 km from the dam, and the stingrays (n=8) were captured by a fishing net and observed at the bottom of the river. Of these, a pregnant female was killed with paddles and five stillborn pups were expelled with the help of a fisherwoman. In the municipality of Araçatuba, the stingrays (n=3) were captured with a net and harpoon. One of these was captured near the ravine near a local bridge and was a pregnant female that expelled three stillborn pups. In Santo Antônio do Aracanguá, stingrays (n=2) were caught by hooks and fishing nets. In the municipality of Pereira Barreto, the stingrays (n=2) were seen on two occasions, on the bank of the ravine and in the riverbed near the port of Pereira Barreto, respectively. Another river stingray (n=1) was captured with a net; however, it was not possible to verify the location of the municipality, which was identified only as the Tietê River. Figure 3 shows the images of the stingrays collected during this period.

It was not possible to identify the species, even with the images of the stingrays, when there were some atypical and hybrid patterns, but the species identified in previous studies were *Potamotrygon motoro* ^1^ ^,^ ^15^ (a gray or brown back of the disc, with the presence of large tricolor ocelli distributed throughout the disc, usually composed of a yellow spot in the center, an orange ring in the middle, and a black peripheral ring) and *Potamotrygon falkneri* ^15^ (dark brown dorsal disc coloration, with light circular spots or orange, oval, vermicular, and/or rosettes spots. The spots were equal to or smaller than the ocular diameter).

## DISCUSSION

Freshwater stingrays colonized a large part of the lower Tietê River region, traveling 130 km after the first capture in the municipality of Itapura over a period of 11 years, reaching downstream of the dam of the Nova Avanhandava Hydroelectric Power Plant in the municipality of Buritama. From 2018 onwards, the number of stingrays caught with fishing nets increased. In this study, only the points with the location and image of the specimen were mapped; however, considering the fishermen's reports about fishing without images, the authors found it important to represent them. There were 16 points with a recorded image of the stingrays and 24 reports without images. Therefore, there were 40 points in the lower Tietê region that were observed and/or included the capture of stingrays. It is possible that this amount was much higher. It was observed that the female could generate up to five offspring and, with no predators, all the offspring reached the adult stage.

The mapping showed that a greater number of stingrays were captured in the municipality of Buritama, probably due to the narrowing of the river in this region that precedes the dam of the Avanhandava Hydroelectric Power Plant. The riverbed is large, more than 3 km from one bank to the other and is known as the “freshwater sea”^13^.

In the municipality of Novo Horizonte, which belongs to a sub-basin in the middle Tietê River, fishermen were unaware that there were stingrays in the river, similarly to the fishermen between the municipalities of Pereira Barreto and Buritama who claimed that river stingrays only existed in the Paraná River before 2016.

**FIGURE 4:**
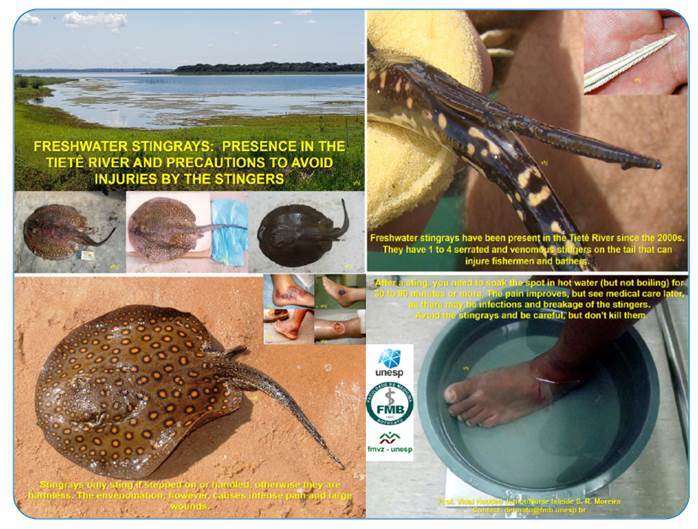
Information leaflet used in campaigns under development in the region.

No stingray injury in humans has been identified in the Tietê River; however, artificial beaches are leisure sites for the population and have considerable tourist economic potential. Along the entire length of the lower Tietê, there are beaches that can cause numerous stingray-related injuries to bathers in the future (Figure 4). Stingrays are migrating upwards in the Tiete River and both the population and fishermen should be alert.
